# Local Health Departments’ Activities to Address Health Disparities and Inequities: Are We Moving in the Right Direction?

**DOI:** 10.3390/ijerph13010044

**Published:** 2015-12-23

**Authors:** Gulzar H. Shah, John P. Sheahan

**Affiliations:** Jiann-Ping Hsu College of Public Health, Georgia Southern University, Statesboro, GA 30460, USA; js02904@georgiasouthern.edu

**Keywords:** health disparities, community health assessment, community health improvement plan, strategic planning, local health departments, health inequities

## Abstract

*Context*: Health disparities are among the critical public health challenges. *Objectives*: To analyze the extent to which local health departments (LHDs) perform activities for addressing health disparities, changes in proportion of LHDs’ performing those activities since 2005, and factors associated with variation in such engagement. *Methods*: We used the 2013 National Profile of LHDs Survey to perform Logistic Regression of activities LHDs performed to address health disparities. *Results*: About 20 percent of LHDs did not perform any activity to address health disparities. Significant decreases occurred since 2005 in the proportion of LHDs that performed health disparity reduction/elimination activities for four activities. LHD characteristics significantly associated (*p*≤0.05) with the increased likelihood of performing activities to address health disparities were: recent completion of community health assessment, community health improvement plan and agency wide strategic plan. Other significant positive impacts on such activities included per capita expenditures, local governance, having one or more local boards of health, larger population size and metropolitan status of the LHD jurisdiction. *Conclusions*: Reduced infrastructural capacity of LHDs has resulted in fewer LHDs addressing health disparities in their jurisdictions. LHD characteristics associated with higher performance of activities for health disparity reduction identified by this research have important policy implications.

## 1. Introduction

According to Braveman, health disparities are differences in the quality of healthcare and health status among population subgroups based on race, ethnicity, sexual orientation, immigration status, age, and socioeconomic status [1], and they represent a persistent public health problem [1,2,3,4,5,6,7,8]. Health disparities can lead to differing outcomes and quality of life among individuals with different demographic backgrounds [2,3,4,5,6,7,8,9,10,11,12]. Health disparities exist in the United States, in various forms and across various subgroups, including race/ethnicity categories, age, gender, sexual orientations and other social segments in regards to inequalities in screening, rates of diseases contracted, inequities in mortality, and a lack of legislative and judicial power [13,14,15,16,17,18,19,20,21,22,23]. Various national initiatives have tried to garner awareness about the existence of racial and social disparities in the United States. National plans, such as Healthy People 2020, are a call to action for reducing health disparities by addressing social determinants of health among disadvantaged populations.

In order to address the persistent problems of disparities in health, local health departments (LHDs) are expected to proactively identify such disparities in their communities by conducting community health assessments (CHA), preparing an agency-wide strategic plan (CHIP) that is reflective of CHA, and address these disparities by developing a strategic plan (SP) [24,25,26,27,28]. LHDs who undergo these activities are potentially more able to understand and address health disparities in their jurisdictions, because they have data and evidence about the existence of the disparities and the population groups affected by them [26,27]. With an increasing emphasis on identifying upstream root-causes of public health issues by focusing on social determinants of health, it is critical to look at these issues from a broad public health perspective concerning social determinants of health due to the far reaching health disparities between various demographic and social groups in the United States [11,12,29]. Multiple factors influence health disparities such as access to care, socio-economic factors, the physical environment, and education [2].

LHDs are at the vanguard in the fight to eliminate disparities in the population [30], and they are strategically positioned to address this issue as they represent the frontline of public health. Using the National Association of County and City Health Officials (NACCHO) Profile Study data, we can estimate their ability and activities to address health disparities in their communities. Additionally, because they have intimate knowledge of their communities they are in a better position than other types of health agencies to eliminate disparities specific to their jurisdictions [30,31,32,33,34].

While various national initiatives (e.g., accreditation of public health agencies through Public Health Accreditation Board or PHAB) underscore the need to address health disparities, it is important to gauge the level of effort and strategy at the local level, given that reduced staff and budgets may have affected LHDs’ infrastructure and abilities to address health disparities [32,33,34,35,36]. LHDs can make some difference by working with strategic partners in reducing the inequities leading to disparities or assuring access to health care or by directly providing primary care on a limited scale, particularly to underserved and disenfranchised population subgroups [35,37].

There appears to be significant gaps in the literature concerning how local health departments address health disparities. The objective of this study is to examine and fill in knowledge gaps concerning LHDs, specifically by analyzing (1) the extent to which LHDs engage in strategies and activities for addressing health disparities and (2) variation in involvement of LHDs in specific activities in their efforts to address health disparities in their communities. Such sub-group comparisons are significant because important differences exist in health issues and needs across LHDs.

## 2. Methods

### 2.1. Study Design and Sample

We used three waves of the National Profile of LHDs (Profile) Study for which survey data were collected by National Association of County and City Health Officials (NACCHO), using census approach and web-based structured questionnaires, administered to all LHDs meeting NACCHO’s definition of a local health department. Each of these surveys used a cross-sectional design, census approach and a comprehensive set of questions to elucidate numerous aspects of LHDs’ infrastructural capacities and services, with response rates of 80%, 83%, and 79% respectively in 2005, 2008, and 2013 [38,39,40,41]. While the questions on LHDs’ engagement in activities to reduce disparities were administered to all LHDs in the 2008 Profile survey, these questions were administered to only sub-samples of 519 LHDs in the 2005 Profile survey and 625 LHDs in 2013 with response rates of 81 percent in both studies [38,41]. These samples were selected using stratified random sampling without replacement using seven strata defined by the size of the population in the LHD jurisdiction. The multivariable analyses were conducted using the 2013 survey because pooling of the data from multiple years was not possible due to variation in sampling designs across survey years.

### 2.2. Measures

Our primary measure of interest was the extent to which LHDs in the United States engage in activities to address health disparities in the past two years. In 2005, LHDs were provided the following definition of inequities, “Health inequity is a phrase used to describe a difference in the health of groups in the population that is both avoidable and unfair. In the United States, health inequities exist for almost all conventionally measured health outcomes. The causes of health inequities include racism and poverty as well as differences in the quality of living environments, including homes, food, air, water, jobs, education, and transportation”. LHDs were then asked to “Check each activity that your LPHA has done in the past three years”: (1) “Describing health disparities in your jurisdiction using data”; (2) Conducting original research that links health disparities to differences in social or environmental conditions; (3) Educating elected or appointed officials about health disparities and their causes; (4) Training your workforce on health disparities and their causes; (5) Recruiting workforce from communities adversely impacted by health disparities; (6) Prioritizing resources and programs specifically for the reduction in health disparities; (7) Taking public policy positions on health disparities (through testimony, written statements, media, *etc.*); and (8) Supporting community efforts to change the causes of health disparities.

In the 2008 Profile Survey, disparities were defined as, “Health disparities can be defined as differences in health status that occur among population groups”, and LHDs were asked to “Check each activity that your LHD has done in the past two years to address health disparities” with the same activity list of eight activities used in 2005. In the 2013 Profile Survey, the definition of health disparities and the questions remained the same but an additional activity was included in the list. In addition to the ones included in the 2005 and 2008 surveys; *offering staff training in cultural/linguistic competency* was included in the 2013 Profile Survey. Each of these activities resulted in dichotomous variables, coded as checked (or yes), or un-checked (or no). For the multivariable analyses (Logistic Regression Models), we selected 6 activities for the analyses, excluding the three least commonly performed activities. The least performed activities were not included in the multivariable analyses because they presented the risk of small and zero cells in those analyses, as they would have increased the risk of chance variation.

The *independent variables* included those characteristics of LHDs and LHD leadership theoretically or empirically linked with LHD’s knowledge about existence of disparities in their jurisdiction [24], or infrastructural capacities [9,24,30,31], enabling them to perform activities to address disparities. Population of LHD jurisdiction (in 10,000) and *per capita* expenditures (coded as, not reported; quintiles, 1st < $19; 2nd $19–$29; 3rd $30–$46; 4th $46–$75; 5th ≥ 76), reflected infrastructural capacity and economies of scale known to effect similar performance of public health functions by LHD [41]. Completion of community health assessment (CHA), community health improvement plan (CHIP) and completion of strategic plan (SP) were coded as “completed within 5 years”, and “No/Not within 5 years AND no plan in the next year”, to match PHAB requirement of completion of these pre-requisites within 5 years to be considered current [42]. The CHA, CHIP and SP are theoretically relevant in that they are instrumental in identification of health disparities and formal prioritization. Number of information systems implemented by LHDs, was operationalized using the question, “Indicate your LHD’s level of activity for each of the following health informatics areas”. The question included the following informatics areas, and the variable was formulated by counting the number implemented, (1) Electronic Health Records (EHRs); (2) Health Information Exchange (HIE); (3) Immunization Registry (IR); (4) Electronic Disease Reporting System (EDRS); (5) Electronic Lab Reporting (ELR); and (6) Electronic syndromic surveillance system. Information systems can be instrumental in assistance with identification of disparities (e.g., immunization systems can help identify disparities in completion of immunization). LHDs’ governance was reflected by two variables, having one or more *local boards of health vs.* no local boards of health, and a governance category that reflects the centralization of LHD governance relative to the state health department. Presence of local boards of health (LBOH) also reflected LHDs’ connectivity and their governing body’s exposure to diverse perspectives. The metropolitan *vs.* non-metropolitan status of the LHD jurisdiction was created based on the NCHS definition for urban-rural counties. For LHDs not consisting of single county or those consisting of other complex structures, NACCHO’s GIS data table [43] was used to designate metropolitan (when all of the geographic units were metropolitan), non-metropolitan (when all of the geographic units were non-metropolitan), predominantly metropolitan (when metropolitan areas of the LHD had greater population than non-metropolitan), and predominantly non-metropolitan (when non-metropolitan areas of the LHD had greater population than metropolitan).

### 2.3. Analysis

We performed all analyses for this study using SPSS Statistics version 23 (IBM Corporation, Armonk, NY, USA). In order to compute estimates that are generalizable to all LHDs, we accounted for sampling design and disproportionate response rate by population category, by using statistical weights computed for Module 3 for 2005 data, Core set of questions for 2008, and Module 2 for 2013. To highlight the distribution of LHD characteristics we computed descriptive statistics for the categorical variables and means and standard deviation for the continuous variables. For the multivariable analysis, we computed six separate logistic regression models, with each activity as the dichotomous dependent variable.

## 3. Results

Table 1 shows the descriptive characteristics of the LHDs in the study. As indicated in Table 2, there was a significant decrease in the number of LHDs’ activities intended to reduce health disparities from 2005–2013. In this time period, the percentage of LHDs engaging in educating elected or appointed officials about health disparities and their causes decreased significantly from 55.5% in 2005 to 44% in 2013. The proportion of LHDs recruiting workforce, from communities adversely impacted by health disparities decreased from 24.8% (2005) to 17.8% (2013). LHDs engaged in prioritizing resources and programs specifically for the reduction in health disparities decreased from 50.2% (2005) to 33.6% (2013). Lastly, the percentage of LHDs taking public policy positions on health disparities decreased from 26.7% (2005) to 15.8% (2013).

**Table 1:** Percent distribution of the LHDs in the sample by infrastructural, governance and other characteristics, 2013 National Profile of LHDs Survey.

	N (un-Weighted)	Percent (Weighted)
Per Capita Expenditures (Quintiles rounded to whole dollar)		
<$19	87	16.7
$19–$30	75	13.8
$31–$45	74	14.5
$46–$75	76	14.7
$76 or more	61	12.1
Not Reported	132	28.2
CHA completed within 5 years		
Yes, within 5 years	363	71.6
No but plan to in the next year	55	12.0
No/Not within 5 years AND no plan in the next year	78	16.4
CHIP completed within 5 years		
Yes, within 5 years	278	54.3
No but plan to in the next year	114	23.1
No/Not within 5 years AND no plan in the next year	104	22.7
SP completed within 5 years		
Yes, within 5 years	239	47.3
No but plan to in the next year	116	22.7
No/Not within 5 years AND no plan in the next year	138	30.0
Type of governance		
Decetralized (local governance)	405	79.5
Centralized (*i.e.*, state governance)/shared	100	20.5
LHD has 1 or mroe LBOH		
No	150	28.9
Yes	345	71.1
Metropolitan status of the LHD jurisdiction		
Metropolitan		36.1
Non-metropolitan)	236	63.9
	**Number**	**Mean (variance)**
Population (in 100,000 s)	505	1.247 (13.695)
Number of information systems (ELR, HIE, IR, EDRS, ELR, ESS) implemented by LHD	493	3.044 (2.036)

**Table 2:** Percent of LHDs conducting specific activities to address health disparities, 2005, 2008, and 2013.

Activities to Address Health Disparities	2005 (Activities in Past 3 Years)			2008 (Activities in Past 2 Years)			2013 (Activities in Past 2 Years)		
	%	Confidence Interval		%	Confidence Interval		%	Confidence Interval	
		LL	UL		LL	UL		LL	UL
Describing health disparities in your jurisdiction using data	54.9	49.5	60.4	51.5	49.4	53.6	57.2	52.4	61.9
Conducting original research that links health disparities to differences in social or environmental conditions	11.5	8.2	14.8	11.2	9.9	12.4	10.9	8.2	13.7
Educating elected or appointed officials about health disparities and their causes	55.5	**50.1**	**61.0**	45.6	43.6	47.7	44.0	**39.3**	**48.6**
Training your workforce on health disparities and their causes	51.4	46.0	56.8	49.7	47.7	51.8	48.1	43.4	52.8
Recruiting workforce from communities adversely impacted by health disparities	25.8	**21.3**	**30.2**	20.1	18.5	21.8	17.8	**14.5**	**21.2**
Prioritizing resources and programs specifically for the reduction in health disparities	50.2	**44.8**	**55.6**	39.7	37.7	41.7	33.6	**29.2**	**37.9**
Taking public policy positions on health disparities (through testimony, written statements, media, *etc.*)	27.7	**23.1**	**32.4**	20.2	18.6	21.8	15.8	**12.5**	**19.0**
Supporting community efforts to change the causes of health disparities	62.3	56.9	67.7	58.4	56.4	60.5	54.4	49.7	59.1
None of the above activities	20.9	16.2	25.7	22.0	20.3	23.8	16.0	12.3	19.6
Offering staff training in cultural/linguistic competency	X	X	X	X	X	X	47.3	42.6	52.0

Notes: the confidence bounds in bold indicate a statistically significant change from 2005 to 2013 at *p* ≤ 0.05; X in the last row indicates that data were not collected on this item in the particular year. Abbreviations: LL, lower limit of the confidence interval; UL, Upper limit of the confidence interval.

Results of our multivariable logistic regression models (Table 3) show that *per capita* expenditure had, for the most part, a significant positive association with all of the dependent variables except offering staff training in cultural/linguistic competency. For most of the activities, LHDs with higher *per capita* expenditures had higher odds of performing the activities to reduce health disparities. Having completed a CHA within five years (*vs.* no and no plans for next 12 months) was positively and significantly associated with activities that addressed health disparities in all of the categories except training LHDs’ workforce. 

**Table 3:** Logistic regression of activity performed by LHDs in the past two years to address health disparities, defined as differences in health status that occur among population groups.

LHD Characteristics	Describing Health Disparities in Your Jurisdiction Using Data		Supporting Community Efforts to Change the Causes of Health Disparities		Training Your Workforce on Health Disparities and Their Causes		Offering Staff Training in Cultural/Linguistic Competency		Educating Elected or Appointed Officials about Health Disparities and Their Causes		Prioritizing Resources and Programs Specifically for the Reduction in Health Disparities	
	AOR	*p*	AOR	*p*	AOR	*p*	AOR	*p*	AOR	*p*	AOR	*p*
Per Capita Expenditures (*vs.* <$19)		**0.000**		**0.000**		**0.000**		0.539		**0.000**		**0.000**
Not reported	1.430	**0.038**	1.882	**0.000**	0.453	**0.000**	1.202	0.254	0.914	0.580	1.175	0.334
$19–$30	4.379	**0.000**	1.329	0.084	0.957	0.777	0.958	0.790	2.902	**0.000**	1.809	**0.000**
$31–$45	1.096	0.593	0.699	**0.029**	0.779	0.112	1.034	0.835	0.692	**0.025**	0.852	0.342
$46–$75	1.925	**0.000**	0.695	**0.037**	0.539	**0.000**	1.163	0.371	1.351	0.076	1.156	0.406
$76 or more	1.419	**0.033**	0.938	0.683	0.962	0.797	0.942	0.696	1.429	**0.019**	0.755	0.089
CHA completed within 5 years (*vs.* No/Not within 5 years AND no plan in the next year)		**0.000**		0.000		0.467		**0.000**		**0.000**		**0.014**
Yes, within 5 years	2.989	**0.000**	1.653	**0.006**	1.236	0.229	2.334	**0.000**	1.277	0.180	0.625	**0.021**
No but plan to in the next year	1.348	0.196	0.883	0.584	1.145	0.534	0.840	0.450	0.463	**0.001**	0.485	**0.004**
CHIP completed within 5 years (*vs.* No/Not within 5 years AND no plan in the next year)		**0.032**		**0.006**		**0.000**		**0.046**		**0.000**		**0.000**
Yes, within 5 years	1.558	**0.009**	1.540	**0.010**	1.997	**0.000**	1.238	0.198	1.425	**0.040**	2.722	**0.000**
No but plan to in the next year	1.405	**0.068**	1.798	**0.001**	2.070	**0.000**	1.556	**0.017**	2.318	**0.000**	2.935	**0.000**
SP completed within 5 years (*vs.* No/Not within 5 years AND no plan in the next year)		**0.000**		**0.000**		**0.000**		**0.001**		**0.000**		**0.000**
Yes, within 5 years	3.378	**0.000**	2.216	**0.000**	2.219	**0.000**	1.454	**0.001**	1.649	**0.000**	1.579	**0.000**
No but plan to in the next year	2.225	**0.000**	2.503	**0.000**	1.480	**0.003**	1.012	0.932	2.413	**0.000**	1.823	**0.000**
Local governance (*vs.* state/shared governance)	1.618	**0.001**	1.233	**0.120**	1.530	**0.001**	1.861	**0.000**	0.871	0.294	0.946	0.700
LHD has 1 or more LBOH (*vs.* None)	1.283	**0.041**	1.389	**0.004**	1.254	**0.038**	1.125	0.288	1.330	**0.010**	1.308	**0.023**
Population (in 100,000 s)	1.242	**0.000**	1.607	**0.000**	1.116	**0.000**	1.096	**0.000**	1.040	**0.030**	1.105	**0.000**
Number of information systems (ELR, HIE, IR, EDRS, ELR, ESS) implemented by LHD	1.208	**0.000**	1.071	**0.044**	1.033	0.314	1.364	**0.000**	1.221	**0.000**	1.273	**0.000**
Metropolitan status of the LHD jurisdiction (*vs.* non-metropolitan)	1.707	**0.000**	0.938	0.592	0.948	0.616	1.112	0.320	1.546	**0.000**	1.257	**0.038**

Abbreviations: AIRR, Adjusted Incident Rate Ratio; EHR, Electronic Health Records; HIE, Health Information Exchange; IR, Immunization Registry, EDRS, Electronic Disease Reporting System; ELR, Electronic Lab Reporting; ESS, Electronic disease surveillance; CHA, community health assessment; CHIP, community health improvement plan; SP, Strategic Plan; CI, confidence interval; LHD, local health department; LBOH, Local Board of Health; Bold values indicate that the subgroup differences are significant at *p* ≤ 0.05.

Having completed a CHIP within five years (*vs.* no and no plans for next 12 months) significantly increased the odds of performing all six activities to address health disparities, including describing health disparities using data (AOR, 1.56), supporting community efforts to change the causes of health disparities (AOR, 1.54), training your workforce on health disparities and their causes (AOR, 2.00), offering staff training in cultural/linguistic competency (AOR, 1.24), educating elected or appointed officials about health disparities and their causes (AOR, 1.43), and prioritizing resources and programs specifically for the reduction in health disparities (AOR, 2.72). Having completed a strategic plan within 5 years (*vs.* no and having no plans for next 12 months) was also significantly associated with increased odds of performing all six activities—describing health disparities (AOR, 3.38), supporting community efforts (AOR, 2.10), training your workforce (AOR, 2.10), offering staff training (AOR, 1.45), educating elected or appointed officials (AOR, 1.65), and prioritizing resources and programs (AOR, 1.58). 

Odds for health disparity prevention activities for LHDs with decentralized (*i.e.*, local) governance (*vs.* centralized/state or shared governance) were significantly higher in three of the six categories—describing health disparities (AOR, 1.62), training your workforce (AOR, 1.53), and offering staff training (AOR, 1.86). The size of population in LHD jurisdiction was also positively associated with all six of the dependent variables, including describing health disparities (AOR, 1.24), supporting community efforts (AOR, 1.61), training your workforce (AOR, 1.12), offering staff training (AOR, 1.10), educating elected or appointed officials (AOR, 1.04), and prioritizing resources and programs (AOR 1.11). LHD with one or more LBOH (*vs.* none) had significant higher odds of performing all activities (except offering staff training in cultural/linguistic competency), including describing health disparities (AOR, 1.28), supporting community efforts (AOR, 1.39), training LHD workforce (AOR, 1.25), educating elected or appointed officials (AOR, 1.33), and prioritizing resources and programs (AOR, 1.31). Superior informatics capacity of LHDs improved their odds of performing activities to address health disparities, including describing health disparities (AOR, 1.21), supporting community efforts (AOR, 1.07), offering staff training (AOR, 1.36), educating elected or appointed officials (AOR, 1.22), and prioritizing resources and programs (AOR, 1.27). Lastly, the metropolitan status of a LHD (*vs.* non-metropolitan) was significantly associated with increased odds of three activities—describing health disparities (AOR, 1.71), educating elected or appointed officials (AOR, 1.55), and prioritizing resources and programs (AOR, 1.25).

## 4. Discussion

Some of LHDs’ activities to address disparities declined in the year 2008 and further declined in 2013, compared with 2005. This is noteworthy, given that an increase was expected in the proportion of LHDs engaging in such activities, in the post Affordable Care Act landscape characterized by efforts to improve population health through prevention and improved access to care among traditionally underserved population. It is also of note considering numerous national bodies (e.g., PHAB) recommending that public health agencies ought to tackle health disparities by reducing inequities in social determinants of health. Of particular note is our finding that shows a reduction in frequency of LHDs performing activities concerning public policy, such as taking public policy positions on health disparities (through testimony, written statements, media, *etc.*), or educating elected or appointed officials about health disparities and their causes.

Health disparities result primarily from socio-political and economic inequities (social determinants of health) among population subgroups based on race/ethnicity, gender, age, immigration status, sexual orientation, and other individual or subgroup characteristics [44,45]. Having fewer LHDs informing activities to educate elected or appointed officials about health disparities and their causes has the potential to influence policies concerning a multitude of socio-political and economic factors that can impact health disparities. Reduction in the proportion of LHDs engaged in recruiting workforce from communities adversely impacted by health disparities is perhaps more clearly indicative of impact of 2007–2008 recession on LHDs’ activities [46]. Overall the decline in workforce and repeated budget cuts year after year [34] may have reduced LHDs’ ability to recruit more staff in general, which in turn might have resulted in a reduction in this activity. Decline in post-recession budgets is perhaps also the reason for LHDs’ inability to prioritize resources and programs specifically targeting reduction in health disparities [46].

Our results from *multivariable analyses* also have important implications for public health practice and policy. Positive association between *completing a CHA*, -—with all but one dependent variable is indicative of the value of data-driven public health practice. In order to consider strategies for addressing health disparities, LHDs must know the specific health indicators for which disparities exit and CHA can be instrumental in identifying those specific health disparity indicators [23]. Completion of *CHIP* is also positively associated with an LHD’s ability to administer programs addressing health disparities. PHAB standards require LHDs to sequence CHA and CHIP so that their CHA must inform their CHIP, which means that priority health issues highlighted in CHA get incorporated into CHIP [27]. This explains the association of CHIP and health disparities. An *agency-wide strategic plan* (SP) helps an agency outline their future strategic goals and initiatives. Without a proper well thought out SP an agency may not be able to fully utilize all of its resources. Therefore, LHDs with a SP that is properly informed by CHA and CHIP are likely to designate addressing health disparities a high strategic priority if health disparities are highlighted in their CHA and CHIP [47].

*Decentralized governance* (as opposed to state or shared governance)—was associated with a LHDs’ ability to *describe disparities*, *training their workforce*, and *offering staff assistance*. *Centralized governance* of the LHD—may make it easier for the state (as umbrella) agency to facilitate these activities for LHDs, lessening LHDs’ need to directly engage in these activities. The population size in a LHD’s jurisdiction is among the strongest factors influencing LHDs’ engagement in activities to address health disparities, perhaps due to economies of scale and scope. A larger size population in a LHD jurisdiction was significantly associated with greater likelihood of performing all six activities to address health disparities. LHDs with higher population may have more resources and lower unit cost to assess the existence of health disparities in their jurisdiction and if needed, formulate strategies to address health disparities [48].

The health informatics capacity of LHDs- was also positively associated with LHDs’ engagement in activities to curb health disparities. The number of *information systems* a LHD possesses is significant in four out of the 6 dependent variable categories. Health *informatics capacity* can increase LHDs’ ability to collect and communicate disparities-related data, improving their prospects for conducting activities to address health disparities.

Currently, most of the activities LHDs perform to combat health disparities focus on disparities and some of their proximate determinants (e.g., showing the overall overlay between poverty and health outcomes), but a shift in focus is needed in identifying upstream factors and inequities responsible for creating and sustaining economic and health disparities. For instance why is the life expectancy 10 years apart in neighborhoods that are two miles apart?

To tackle health disparities adequately, instead of focusing on health disparities as outcomes for the population, LHDs’ may want to consider partnering with other institutions and critically evaluate institutional practices that create inequities. LHDs’ strategies and activities that examine decisions of social institutions and how the integration of these decisions into institutional practices create inequities and social injustices can be effective in preventing health disparities. Highlighting these inequities and working with other stakeholders to bring about policy and practice changes in the respective institutions can be very fruitful.

This might mean simple things, such as completion of CHA by LHDs not only looking at the health outcomes and racial/ethnic disparities with respect to those outcomes but also looking at the data that will identify the root causes of disparities. We see from the results that completing a CHA and a CHIP were significantly associated with a LHDs performing activities to reduce health disparities. Thus, we can see that encouraging LHDs to engage in these activities could increase their efforts to address inequities in their jurisdictions. Enabling better federal and state policy to encourage the use of CHA, CHIP, and SP in LHDs could increase LHDs effort to reduce disparities in their communities, some of which the NACCHO profile study has shown to be decreasing since 2005. When patterns of inequalities are indicated by the CHA data, LHDs, in preparing the community health improvement plan (CHIP), should not simply assume that these differences are merely bad luck, or unfortunate events, or caused by people’s individual behavior. They must try to explain the patterns as resulting from historic and/or ongoing policies and institutional practices that created these inequities over time, not necessarily intentionally, but because people’s needs were ignored for the sake of other competing interests.

## 5. Limitations

Our study has several limitations, hence our findings should be interpreted within this context. First, the Profile study, the source of secondary data for our analysis, comprises a broad range of topics on public health practice and infrastructural capacities. This study is not, however, designed to provide exhaustive data on all of the topics. The future research may use qualitative or mixed methods approach to ask more critical and detailed questions. Secondly, the questions included in the Profile study deal with health disparities, which highlight the end product of health inequalities. In order to attack the root cause of disparities and prevent them from happening in the first place, future studies may lay emphasis on institutional policies, norms, and social structures responsible for creating structures of inequitable access to social determinants of health. Thirdly, as with most of the secondary qualitative data, the self-reported information was not independently verified. Finally, the unit of sampling and observation for the Profile study is individual LHD, but the LHDs included in the study have a lot of flexibility regarding who completes the survey. In some LHD’s Profile surveys the questionnaire is completed by LHD top leadership, while in other LHDs programmatic staff complete the survey, and yet multiple staff members from different functional units complete the survey in some others. This variation in level of staff may have resulted in variation in respondents’ understanding of the term (health disparities) and their knowledge about specific strategies to deal with health disparities by the LHDs, resulting in some systematic bias. Additionally, different factors may influence different types of disparities and the way a LHD is able to address them, might be different as well. Lastly, the type of health disparity present varies by the community served. Many factors including population, socio-economic status, environment, *etc.* can affect the type of health disparity present in that community. This could influence the measurement of health disparities.

## 6. Conclusions

Health disparities, an outcome of socio-political and economic inequities, constitute a critical public health issue but some LHDs seem to have experienced a reduction in their capacity to address health disparities. A significant reduction in the proportion of LHDs performing certain activities that can potentially address health disparities implies that LHDs need to recover from recent resource shortages after the budget cuts and staff reductions associated with recent recession of 2007–2008. LHDs lacking the scale and scope required for engaging in activities targeting reduction in health disparities may consider cross-jurisdictional resource sharing as a strategy to build economies of scale. Increase in LHDs’ ability to engage in activities to address health disparities associated with their informatics capacity is among many other potential benefits of the health information technology and information systems. Policies aimed at improving capacity of public health systems to address disparities should consider investments in building robust informatics capacity of LHDs. The benefits of completing CHA, CHIP and SP (the three pre-requisites of PHAB accreditation) are also highlighted by our research. Future research should collect primary data on the extent to which LHDs engage in tackling health disparities adequately by collecting qualitative data on institutional practices that create inequities.
